# Monetizing the Burden of Childhood Asthma Due to Traffic Related Air Pollution in the Contiguous United States in 2010

**DOI:** 10.3390/ijerph18157864

**Published:** 2021-07-25

**Authors:** Minaal Farrukh, Haneen Khreis

**Affiliations:** 1 Center for Advancing Research in Transportation Emissions, Energy and Health (CARTEEH), Texas A & M Transportation Institute (TTI), College Station, TX 77843, USA; Minaal99@tamu.edu; 2 Texas A & M School of Public Health, College Station, TX 77843, USA; 3 MRC Epidemiology Unit, University of Cambridge School of Clinical Medicine, Institute of Metabolic Science, Cambridge Biomedical Campus, Cambridge CB2 0SL, UK

**Keywords:** air pollution, asthma, cost of illness, traffic emissions, public health, economics, pediatric asthma, traffic related air pollution

## Abstract

Background: Traffic-related air pollution (TRAP) refers to the wide range of air pollutants emitted by traffic that are dispersed into the ambient air. Emerging evidence shows that TRAP can increase asthma incidence in children. Living with asthma can carry a huge financial burden for individuals and families due to direct and indirect medical expenses, which can include costs of hospitalization, medical visits, medication, missed school days, and loss of wages from missed workdays for caregivers. Objective: The objective of this paper is to estimate the economic impact of childhood asthma incident cases attributable to nitrogen dioxide (NO_2_), a common traffic-related air pollutant in urban areas, in the United States at the state level. Methods: We calculate the direct and indirect costs of childhood asthma incident cases attributable to NO_2_ using previously published burden of disease estimates and per person asthma cost estimates. By multiplying the per person indirect and direct costs for each state with the NO_2_-attributable asthma incident cases in each state, we were able to estimate the total cost of childhood asthma cases attributable to NO_2_ in the United States. Results: The cost calculation estimates the total direct and indirect annual cost of childhood asthma cases attributable to NO_2_ in the year 2010 to be $178,900,138.989 (95% CI: $101,019,728.20–$256,980,126.65). The state with the highest cost burden is California with $24,501,859.84 (95% CI: $10,020,182.62–$38,982,261.250), and the state with the lowest cost burden is Montana with $88,880.12 (95% CI: $33,491.06–$144,269.18). Conclusion: This study estimates the annual costs of childhood asthma incident cases attributable to NO_2_ and demonstrates the importance of conducting economic impacts studies of TRAP. It is important for policy-making institutions to focus on this problem by advocating and supporting more studies on TRAP’s impact on the national economy and health, including these economic impact estimates in the decision-making process, and devising mitigation strategies to reduce TRAP and the population’s exposure.

## 1. Introduction

In the United States (U.S.), asthma is one of the most common chronic diseases, affecting approximately 5,530,131 children and 24,753,379 adults and children in 2018 [1,2]. Asthma presents itself as repeated episodes of wheezing, breathlessness, chest tightness, and nighttime or early morning coughing [3]. An individual’s asthma severity can vary. Asthma severity refers to the intensity of the disease process, and is categorized into two types: intermittent severity and persistent severity [4]. Intermittent severity includes people with asthma that is well-controlled without long-term control medication [4]. Persistent severity includes people with well-controlled asthma with long-term control medications and people with uncontrolled asthma who are not on long-term control medication [4]. Nearly 60% of children with current asthma have persistent asthma and 40% have intermittent asthma [4]. Asthma may develop as a result of various risk factors, including family history, viral respiratory infections, allergies, smoking, obesity, and exposure to environmental contaminants, including traffic-related air pollution (TRAP) [5,6]. This paper focuses on the latter risk factor: the association with TRAP.

TRAP refers to the wide range of air pollutants emitted by traffic through combustion and non-combustion routes and dispersed into the ambient air. TRAP is a major source of ambient air pollution in urban areas and cities [6,7]. Traffic emits pollutants including carbon monoxide (CO), nitrogen dioxide (NO_2_), nitrogen oxides (NO_x_), particulate matter with a diameter less than 2.5 micrometers (PM_2.5_), particulate matter with a diameter less than 10 micrometers (PM_10_), benzene, lead, and sulfur, and others [7], and leads to the formation of secondary by-products and aerosols (e.g., ozone (O_3_) and nitrates). Other pollutants include organic and chemical material from tire wear, brake wear, and resuspended dust [8]. In this paper, we focus on the cost impacts of childhood asthma caused by NO_2_, as it is a pollutant which is relatively specific to TRAP in urban areas and cities [9].

Air pollution is an environmental risk factor that was proven to exacerbate pre-existing asthma cases, but in the past, it was not believed to lead to the onset of new asthma cases [10,11,12]. However, an increased understanding of interactions between genetic and environmental factors, and an ever-growing evidence base have shown that TRAP exposure may lead to the development of asthma in children [7,9,13,14]. As emerging evidence shows the effect TRAP can have on asthma incidence, it is important to understand how expansive that effect is. The impact of TRAP on childhood asthma in the U.S. has been quantified by a 2019 study, which estimated the number and percentage of childhood asthma incident cases attributable to TRAP in 2000 and 2010 [15]. Th study concluded that, on average, the percentage of childhood asthma incident cases attributable to TRAP in the contiguous U.S. may range between 18% (NO_2_) and 42% (PM_10_), depending on the pollutant selected to represent the TRAP mixture, with NO_2_ being more specific [15]. These results indicated that TRAP may be responsible for a large number of preventable childhood asthma cases [15]. Another study in 2020 quantified the number of childhood asthma incident cases attributable to NO_2_ in the U.S., citing NO_2_ as a more specific marker of the TRAP mixture [16]. Using state-level incidence rates, Khreis et al. [16] estimated that a total of 134,166 (95% CI: 75,177–193,327) childhood asthma incident cases were attributable to NO_2_, accounting for 17.6% of all childhood asthma incident cases [16]. Khreis et al. [17] also estimated that between 7% to 12% of all childhood asthma cases in Bradford, United Kingdom were attributable to TRAP [17].

Living with asthma can carry a huge financial burden for individuals and families. Direct costs such as costs for alternative treatment, medications, primary care consultations, hospital emergency and outpatient attendance, ambulance and other transportation, and hospital admissions, in addition to indirect costs such as missing school and workdays, contribute to asthma’s financial burden [18]. In 2007, asthma resulted in more than $56 billion in medical costs, lost school and workdays, and early death in the U.S. for all cases [19]. In 2013, the number of asthma-related missed school days was 13.8 million [20]. In 2015, the total number of missed school days among children with asthma ranged from 9020 days to 617,980 days across the 50 U.S. states and Washington, District of Columbia (D.C.) [21]. The cost range by state for school absenteeism ranges from $1.4 million to $116.5 million, while the cost for missed school and workdays ranges from $4.4 million to $344.9 million by state [21]. Furthermore, people with asthma experience over 497,000 hospitalizations annually in the U.S. collectively [22]. The average per person annual medical costs for asthma is estimated to be $3300 for the entire population while the annual medical costs per child were estimated to be $1740 [23]. Notably, costs vary widely by state. The Nurmagambetov et al. [21] study found that the total medical and absenteeism costs for childhood asthma in 2015 ranged from $7.7 million in Wyoming to $488.1 million in California. The total costs (for children and adults) ranged from $65.1 million in Wyoming to $3718.1 million in California [21].

In this study, we focus on estimating the economic burden of childhood asthma incident cases attributable to NO_2_. We will use previous estimates of childhood asthma incident cases attributable to NO_2_ in the U.S. from Khreis et al. [16] to determine its economic impact. While there are previous studies looking at the economic burden of pediatric and adult asthma nationally or by state [21,23,24], few studies specifically address the economic burden of childhood asthma cases attributable to air pollution in the U.S. Those that exist are either conducted in small communities or cities, different countries, address other health issues related to environmental contaminants, or focus on only a few cost variables (e.g., only hospitalization costs) (Table 1). Some previous studies were restrictive in terms of location; for example, they were set in a single county, such as Los Angeles County, or in cities, such as Shanghai, Riverside, and Long Beach [25,26]. One study examined the association between sub-chronic exposure of six outdoor air pollutants (PM_2.5_, PM_10_, O_3_, NO_2_, SO_2_, CO) and pediatric asthma hospitalization length of stay, charges, and costs, but it did not follow a burden of disease (BoD) assessment methodology like this work [27]. The methodology of Roy et al. [27] is also very different than our study, as we built on a previously published BoD assessment and published direct and indirect asthma costs by state. Our study fills a gap in the literature with an overarching assessment of the full economic impacts of childhood asthma incident cases attributable to NO_2_, at the U.S. state level.

Our study is in a unique position to conduct an overarching assessment of the economic impacts of pediatric asthma incidence attributable to NO_2_ in the U.S. We aim to monetize childhood asthma incidence in the U.S. specifically attributable to NO_2_ at the state level, building on the most recent and comprehensive BoD and economic impact assessment work [16,21]. We will calculate both indirect and direct costs and will provide an economic analysis, showing the total cost of childhood asthma incident cases attributable to NO_2_ in each state using the most holistic definitions for the cost of illness. 

## 2. Materials and Methods

### 2.1. Overview

In this study, we examine the economic impact of childhood asthma incident cases attributable to NO_2_. We conducted an economic study by finding the cost of a single childhood asthma case in each state and multiplying it with the number of childhood asthma incident cases attributable to NO_2_ in each state. For our childhood asthma incident cases attributable to NO_2_ counts, we used state estimates which we developed in our previous work reported in Khreis et al. [16]. For our cost numbers, we used the previously estimated costs of a single childhood asthma case in each state as calculated from Nurmagambetov et al. [21]. Our study’s results present a state level economic analysis that shows the cost of a single case of asthma in each state and the total costs for all childhood asthma incident cases attributable to NO_2._

### 2.2. Estimation of Childhood Asthma Incident Cases Attributable to NO_2_

Our state level economic analysis focuses on estimating the state-specific economic impacts of childhood asthma incident cases attributable to NO_2_. Our analysis focuses on one traffic-related air pollutant, NO_2_. To calculate the economic cost, we used BoD estimates of asthma at the state-level as reported in Khreis et al. [16], which is briefly described next.

Khreis et al. [16] analyzed data for the 48 contiguous U.S. states and Washington D.C. (D.C.) for 2010 at the census block level. The total population of children aged 0 to 18 in the census data was 73,690,271, i.e., 24% of the total population. The number of children in rural areas is 13,763,183 (19%), 6,994,464 (9%) in urban clusters, and 52,932,624 (72%) in urbanized areas. The number of attributable childhood asthma cases were estimated at the state level. This study used a concentration response function (CRF) to estimate the association between the risk of childhood asthma onset and the exposure to NO_2_ of 1.05 (95% CI = 1.02–1.07) per 4 µg/m^3^ of NO_2_. The CRF was obtained from a meta-analysis of 20 studies examining the association between exposure to NO_2_ and the risk of developing asthma among children from birth to 18 years of age [9]. Annual NO_2_ concentrations were derived from a land-use regression (LUR) model that incorporates spatial and temporal observed air pollutant data [29]. To estimate the state level incidence rate, this study obtained childhood asthma data from the Behavioral Risk Factor Surveillance System (BRFSS) and the Asthma Call-Back Survey (ACBS) from the years 2006 through 2010 [30,31,32]. In brief, Khreis et al. [16] determined the asthma status of children through the BRFSS and the nested ACBS. To determine the asthma status of children, respondents to the BRFSS were asked “Has a doctor, nurse, or other health professional ever said that the child has asthma?” If the answer was “yes,” the respondent was designated as “ever asthma.” If the answer was “no,” the respondent was designated as “never asthma.” Respondents with children designated as “ever asthma” were requested to participate in the ACBS follow-up. To determine the “incident status” of children, respondents to the ACBS were asked “How old was [name of child] when a doctor or other health professional first said [he/she] had asthma? How long ago was that?” If the answer to the latter part of this question was “within the past 12 months,” the respondent was designated as an “incident asthma. Using these data, in addition to sample sizes, sample weights and estimated population, Khreis et al. [16] provided results of the state-specific incidence rates, different to other previous work such as Alotaibi et al. [15]. These rates represent an average estimate from 2006 to 2010 for the states participating in the ACBS/BRFSS. Overall, there were 32 states that had childhood asthma incidence rates and 41 states that had childhood asthma prevalence rates. The remaining states that did not have an incidence rate were assigned the overall aggregate asthma incidence rate as calculated from all available data [16]. 

To estimate the BoD of incident childhood asthma attributable to NO_2_ exposure, the Khreis et al. [16] study followed the standard BoD assessment methods described in Alotaibi et al. [15] and widely used in the literature. The total number of at-risk children residing in a census block was estimated for each state. This was done by subtracting the total number of children within the census block multiplied by the state-specific prevalence rate from the total number of children within the same census block. Khreis et al. [16] estimated the number of childhood asthma incident cases within each census block by multiplying the state-specific asthma incidence rate by the at-risk children in each census block. Then, the relative risk was calculated for asthma onset due to the exposure difference between the estimated exposure levels from the LUR model (NO_2_ concentration at the centroid of each census block) and a hypothetical no exposure scenario (zero concentration for NO_2_) at each census block [29]. The LUR model uses satellite data and Environmental Protection Agency (EPA) air quality monitor readings of NO_2_ concentrations alongside several covariates to estimate NO_2_ concentrations. The model also incorporates temporal scaling by estimating average monthly monitor readings for 11 consecutive years. The final model used has a relatively high predictive power at unmeasured locations, which was tested using a hold-out cross-validation with good model performance (R^2^ = 0.82); this is comparable with other continental scale NO_2_ LUR models [15,29]. The population-attributable fraction (PAF) was then estimated at each census block. The attributable number of asthma incident cases was estimated by multiplying the PAF with the total number of asthma incident cases in each census block. The attributable number of asthma incident cases for each census block was then summed across the state to obtain the total state attributable number of asthma incident cases [16]. 

Khreis et al. [16] reported that South Dakota had the lowest mean NO_2_ concentration (5.2 µg/m^3^), while D.C. had the highest (26.3 µg/m^3^). Across all states with available data, the overall aggregate childhood asthma incidence rate for the years 2006–2010 was 11.6 per 1000 at-risk children. Montana had the lowest aggregate childhood asthma incidence rate of 4.3 per 1000 at-risk children, followed by Louisiana’s incidence rate of 5.8 per 1000 at-risk children, while D.C. had the highest aggregate childhood asthma incidence rate of 17.7 per 1000 at-risk children, followed by Texas’s incidence rate of 16.6 per 1000 at-risk children. States that did not have an incidence rate available were assigned the overall aggregate asthma incidence rate of 11.6 per 1000 at-risk children.

### 2.3. Review of Sources for Costing across the U.S.

To complete an economic analysis of childhood asthma incident cases attributable to NO_2_, we needed to estimate the cost of a single childhood asthma case in the U.S. To do this, we needed to find data on the cost of each category of spending: hospitalizations, office visits, prescription costs, emergency room costs, etc. We started our search by researching public databases, such as IPUMS [census and survey data/ipums.org] and the Medical Expenditure Panel Survey (MEPS), which provide raw data; however, due to a lack of full public access and incomplete datasets, they were not viable options. Instead, we conducted a database review and a literature review to determine the availability of previously calculated estimations of cost across the different categories of spending. We looked at public databases that had estimated costs from national surveys and presented them for public use. Table 2 summarizes the database review and Table 3 summarizes the literature review. Because this research study began with the intention to provide results from both the national and the state level and in both years 2000 and 2010, both year ranges are included in our data and literature review. However, through our research, our focus was narrowed to the year 2010 and the state level incidence only rather than including national estimates, to offer more spatial resolution in data analyses and presentation.

#### 2.3.1. Database Review

Our database review looked at two sources from the Agency for Healthcare Research and Quality (AHRQ): The Health Care Cost and Utilization Project (HCUP) and MEPS. We reviewed each database for the type of direct or indirect costs they researched, how asthma was defined, the year of the data, whether the database provided state estimates, and if asthma costs for children under 18 were reported.

HCUPnet is a free, online query system based on data from the HCUP. The system provides health care statistics and information for hospital inpatient, emergency department, and ambulatory settings, as well as county-level population-based health care data. We looked for results in 2000 and 2010 at the national and state level, and the category of direct costs (Inpatient or Emergency). HCUPnet did not provide indirect costs. These results are summarized in Table 2 and with more detail in Table S1. A second source consulted was the MEPS Household Component summary tables provided by AHRQ. The MEPS Household Component summary tables provide frequently used summary estimates for the U.S. civilian non-institutionalized population on household medical utilization and expenditures, demographic and socio-economic characteristics, health insurance coverage, access to care and experience with care, medical conditions, and prescribed medicine purchases. This resource allows users to stratify by demographic or socio-economic characteristics and plots are generated from selected data. It provides results for costs by category for the entire population and a combined direct cost number for children under 18 at the national level. This source internally defined asthma and combined the costs numbers with chronic obstructive pulmonary disease when reporting the costs.

This database review allowed us to see results straight from national databases and what the estimated costs were. The database review, along with the literature review described below, was reviewed together to determine which source to extract cost numbers from. The literature review is outlined in Section 2.3.2.

Table 2 summarizes our findings from different databases and details the direct cost estimates, the sample size of each database, the age makeup of the sample, how asthma is defined in each source, and how costs are defined and calculated within each database. Table S1 outlines this information in detail.

#### 2.3.2. Review of the Published Studies for Costing in the U.S.

In addition to the database review described above, we conducted a literature review. The literature review focused on finding published estimates for both direct and indirect costs caused by childhood asthma in the U.S. Our review found seven relevant studies, which are described in detail in Table S2 in the Supplementary Material, and briefly next. Barrett et al. [33] reported the average cost per hospital stay for children aged 2 to 17 in both 2000 and 2010 at the national level. This study used the same surveys from the HCUP results (Table 2). Barnett and Nurmagambetov [34] estimated both the direct and indirect costs of asthma for the U.S. population between 2002 and 2007 nationally. This study estimated the cost for both the entire population and children aged 3 to 19. Wang et al. [35] estimated both the direct and indirect cost per child aged 5 to 17 at the national level. Nurmagambetov et al. [21] reported the direct and indirect costs for children aged 0 to 17 for each state. Nurmagambetov et al. [23] estimated the direct and indirect cost nationally but did not break down the estimates for each event for children as they did for the rest of the population. Sullivan et al. [36] estimated the national direct cost of asthma from children aged 6 to 17. Karaca-Mandic et al. [37] estimated the out-of-pocket medication cost for children under 5 and 5 to 17 with asthma. This literature review revealed the current published cost estimates for childhood asthma in the U.S. Table 3 summarizes the findings of our literature review and Table S2 in the Supplementary Materials outlines in detail the direct and indirect costs of each study, the sample size, the age groups of the children, how asthma was defined, and how cost was defined.

#### 2.3.3. Choosing Our Cost Source

After conducting the two above reviews, we considered which sources provided the best cost estimates for our purposes. We wanted to include both indirect and direct costs for children under the age of 18 at both the national and state level. We anticipated taking different cost information from different sources, but this was not feasible because our source options studied different time frames, different age groups, defined asthma differently, and looked at different categories of direct or indirect costs. Because of this, we simplified our study to only focus on estimating the costs of childhood asthma in the U.S. at the state level and in the year 2010 to correspond with the most recent Khreis et al. [16] study, which offers more spatial resolution in data analyses and presentation. Our selected cost source after reviewing our options above was the Nurmagambetov et al. [21] study. 

### 2.4. Estimation of Costs per Childhood Asthma Case in the U.S.

We used Nurmagambetov et al. [21] as our cost source, as it estimates both the direct and indirect costs for children in the U.S., using the most recent available data at the time of their study (2008–2012 data from MEPS). The study also used other data sources: the 2004 National Nursing Home Survey, National Health Accounts, 2000 and 2010 U.S. Census Bureau, 2012 Current Population Survey, Kaiser Family Foundation, 2012 Medicare Beneficiaries data, 2011 Medicaid Statistical Information Statistics, 2014 Bureau of Labor Statistics (BLS) data, and the 2015 Congressional Budget Office Long-Term Budget Outlook.

To define asthma, Nurmagambetov et al. [21] used the International Classification of Diseases, Ninth Revision, Clinical Modification (ICD-9-CM) Code 493. For direct costs, the study describes the direct costs as the summation of all payments when treating asthma. This includes emergency room visits, office-based medical visits, outpatient hospital visits, and prescription medications by the payer. The study defines indirect costs as the total cost of missed school and workdays. 

In order to estimate the per capita asthma-attributable medical costs, Nurmagambetov et al. [21] used the 2008–2012 MEPS dataset. This dataset describes medical costs as payer’s expenses and represents the total annual sum that a payer spent on an individual’s medical care. The study presented costs from three payer systems: Medicaid, Medicare, and private insurance. Direct costs were estimated for all payers as the total of all medical expenditures spent by all payers, including out-of-pocket payments.

A two-part model was used to approximate annual medical expenditures attributable to asthma: a logistical regression and a generalized linear model was used by Nurmagambetov et al. [21]. The first part was a logistic regression which predicts the probability that a person acquired any medical costs. The second part was a linear model with a log link and a gamma distribution. This part estimated the annual medical costs for those with positive expenditure. This model generated the incremental cost of asthma. The incremental cost of asthma is the difference between the predicted annual medical costs of the person with asthma and the predicted annual medical costs of the same person if the person does not have asthma. This model separated asthma’s impact on medical costs while controlling for other factors that can affect health care costs.

To find state level direct costs, previously generated state level cost adjustments from the restricted-access 2003 MEPS dataset was used [21]. Previously generated state-specific cost adjustments for the states were applied. The state level adjustment factors from the 2003 MEPS dataset were multiplied with the 2012 per person cost estimates from the national model. For each state, Nurmagambetov et al. [21] calculated the total medical direct costs by multiplying the average per person asthma-attributable medical expenditures by the number of people with asthma and combining the costs across age and sex categories within each state and payer category.

In the same study, absenteeism or indirect costs are represented by the cost of missed school or workdays. Nurmagambetov et al. [21] presumed that when a child missed school due to asthma, one of the parents (if both were employed) stayed home with the child, which meant missing a day work. The daily salary of that parent is the value of one day of absenteeism [38,39]. Nurmagambetov et al. [21] used the 2008–2012 MEPS dataset to estimate the number of annual workdays lost per capita (for adults) and schooldays (for children) that were caused by asthma. To predict the annual workdays missed among working adults, a negative binomial model where the dependent variable was the annual workdays and the independent variables were identical to those in the medical cost regressions was used. 

The study used a similar approach to estimate schooldays missed among children. For each state, Nurmagambetov et al. [21] calculated the total number of workdays missed by multiplying the age- and sex-specific number of per capita workdays missed by the corresponding age- and sex-specific number of employed adults with children with asthma. They estimated the value of missed workdays using average daily earnings (including fringe benefits) from the 2014 BLS data.

Nurmagambetov et al. [21] reported the per child (under 18) direct indirect cost in each state. The study did not provide the per person indirect costs, but rather the total in each state. Thus, we took the total cost reported and divided it by the number of children with asthma and employed parents as reported by Nurmagambetov et al. [21]. The divided cost gave us the per child (under 18) indirect cost in each state. 

### 2.5. Cost Calculation

We calculated the total childhood asthma incident case’s cost attributable to NO_2_ at the state level by adding the total direct costs with the total indirect costs to yield the total cost of a childhood incident case attributable to NO_2_ in each state. We used the per person direct cost per state estimated by Nurmagambetov et al. [21] and multiplied it by the number of childhood asthma incident cases attributable to NO_2_ in each state estimated by Khreis et al. [16]. That calculation gave us the total direct medical cost in the U.S. for each state. We then used the total indirect costs estimated by Nurmagambetov et al. [21], which we divided by the total number of children with working parents as explained above. This gave us the per person indirect costs for childhood asthma. We then multiplied the childhood asthma incident cases attributable to NO_2_ from Khreis et al. [16] with both the direct and indirect costs separately to get the total cost of the direct and indirect expenditures in each state. We then added the direct and indirect costs for each state to get the total cost of childhood asthma incident cases attributable to NO_2_ in each state.

## 3. Results

### 3.1. Total Costs

The state with the highest annual cost burden was California with about $24,501,859.84 (95% Confidence Interval (CI): $10,020,182.62–$38,982,261.250), and it had the highest number of NO_2_-attributable childhood asthma incident cases of 19,205 (95% CI: 7854—30,555). The state with the lowest annual cost was Montana with $88,880.12 (95% CI: $33,491.06–$144,269.18), and it had the lowest number of NO_2_-attributable childhood asthma incident cases of 69 (95% CI: 26–112). Table 3 reports the total annual cost of NO_2_-attributable childhood asthma incident cases in the U.S. at $178,900,138.989 (95% CI: $101,019,728.20–$256,980,126.65). This represents 19.8% of the cost of all asthma cases in the U.S., calculated by using the total pediatric asthma cases in each state from Khreis et al. [16] and the per child cost of asthma in each state from Nurmagambetov et al. [21]. Our calculation steps are outlined in Table S3. 

### 3.2. Direct and Indirect Costs Breakdown

The state with the highest annual direct cost burden was California with $16,593,120 (95% CI: $6,785,856–$26,399,520), and the state with the lowest direct cost burden was Montana with $68,586 (95% CI: $25,844–$111,328). The total annual direct cost burden in the U.S. was $129,950,707 (95% CI: $73,785,005–$186,264,165), which represents 72.6% from the total cost of NO_2_-attributable childhood asthma incident cases. The state with the highest annual indirect cost burden was also California with $7,908,739.84 (95% CI: $3,234,326.62–$12,582,741.25), and the state with the lowest annual indirect cost burden was also Montana with $20,294.12 (95% CI: $7647.06–$32,941.18). These trends mirrored trends in the number of NO_2_-attributable childhood asthma incident cases in California (the highest) and Montana (the lowest), while trends in the per child direct and indirect costs differed (see the supplementary excel file, which shows the full calculations). The total annual indirect cost of NO_2_-attributable childhood asthma incident cases was $48,949,431.98 (95% CI: $27,234,723.20–$70,715,961.65), which represents 27.4% of the total annual cost of NO_2_-attributable childhood asthma incident cases (Table 4).

## 4. Discussion

### 4.1. Summary and Comparison to Previous Work

In this article, we monetized childhood asthma incident cases specifically attributable to NO_2_ in the U.S. at the state level. We calculated both the direct and indirect costs, which were then summed to estimate the total cost of childhood asthma incident cases attributable to NO_2_ in each state. We conducted this analysis building on previously published estimates in Khreis et al. [16] and Nurmagambetov et al. [21] of both the attributable pediatric asthma incident cases due to NO_2_ and asthma costs, after conducting a database and literature search for sources on the cost of childhood asthma in the U.S. 

Our estimation shows the estimated cost burden of NO_2_ to be $178,900,138.989 (95% CI: $101,019,728.20–$256,980,126.65). This total represents 19.8% of all pediatric asthma costs in the U.S. It is important to denote that this economic impact only represents NO_2_, which we selected as it is a more specific pollutant to the TRAP mixture and does not represent the cost for other traffic-related related pollutants, which may be higher or lower [15,17].

Our literature review, shown in Table 1, only found one national published study that estimated the cost of pediatric asthma associated with air pollution. However, that study was restricted only to estimating the relation between sub-chronic exposures to air pollutants and hospitalization costs and did not expand to other categories of direct or indirect costs [27]. The methodology of Roy et al. [27] is also very different than our study, as we built on a previously published BoD assessment and published direct and indirect asthma costs by state. Our study fills a gap in the literature with an overarching assessment of the full economic impacts of childhood asthma incident cases attributable to NO_2_, at the U.S. state level.

Previous studies which estimated the cost of asthma associated with or attributable to air pollution were either conducted in small communities or cities, different countries, or focused on only a few cost variables (e.g., hospitalization costs only) (Table 1). Guo and Chen. [26] studied the short-term effect of outdoor air pollution on asthma-related patient visits and their economic costs and calculated the costs of patient visits by each pollutant, i.e., SO_2_, O_3_, CO, PM_2.5_, PM_10_, and NO_2_, in Shanghai, China. The study found that the total loss was 197 million U.S. dollars per year [26]. Brandt et al. [25] estimated the cost of childhood asthma cases attributable to residential near roadway pollution (NRP) in Los Angeles County for regional O_3,_ and NO_2_ in 2007. The study found that the cost of air pollution-related asthma in Los Angeles County in 2007 was $441 million for O_3_ and $202 million for NO_2_ in 2010. These estimates are significantly higher compared to our estimates and are not directly comparable. This may be due to a few reasons. The study by Brandt et al. [25] looked at both the direct and indirect costs of asthma attributable to air pollution, and the direct and indirect costs of an asthma case exacerbation, regardless of the immediate cause of exacerbation. They included causes which are not related to air pollution, and the willingness to pay (WTP) for a household beyond the household’s current expenditures. In addition, Brandt et al. [25] had a higher number of childhood asthma cases attributable to NRP at 27,100 (95% CI, 4900–51,200), making their total cost for asthma cases attributable to air pollution higher compared to our study. This study used the asthma prevalence estimated from a previous study, i.e., Perez et al. [40]. This study also estimated asthma cases attributable to near-roadway and regional air pollution in Los Angeles County using a CRF from the Children’s Health Study that is different from the CRF underlying our work. For their cost calculations, they estimated the direct cost of goods and services and the indirect cost of caregivers’ lost wages. For the direct costs of health care, they used the amount charged rather than the amount paid because amounts charged are not confounded by insurance status. This put their total costs to be $3000 per asthma case and $1500 per a single episode of bronchitis. As such, our estimates are not directly comparable due to the many methodological differences, as outlined above. 

Brandt et al. [28] estimated the cost of childhood asthma cases attributable to TRAP in two Californian cities using proximity to traffic to indicate the TRAP exposure and levels of NO_2_ and O_3_ to estimate the cost of air pollution attributable exacerbations of asthma, following methods similar to Brandt et al. [25]. The authors found that the annual costs of TRAP-attributable cases were U.S. $2,808,300 in Riverside and U.S. $6,120,000 in Long Beach. This study estimated case counts using their previous study, Perez et al. [41], which estimated childhood asthma-related illnesses by deriving population-attributable risk fractions in a BoD assessment framework, including scenario analyses. Compared to our study, Brandt et al. [28] also has higher attributable asthma cases and higher per person asthma costs, although these are not directly comparable, as they pertain to only two communities in California, rather than the whole state. The authors included bronchitis episodes and exacerbations of other-cause asthma cases from air pollutants. Their indirect costs were 3× higher when compared to our study for the state of California specifically, but as highlighted above, there are many methodological differences that hinder comparability and highlight that the total cost may be much higher if the exacerbations of asthma cases, both TRAP and non-TRAP induced, were included in our analyses. However, our scope was different and only focused on estimating the cost of those cases specifically induced by TRAP. While our study fills an important research gap and presents a comprehensive estimate of the cost of asthma in each state and both direct and indirect costs, when compared to current published estimates, our costs may be underestimated.

Another study, Roy et al. [27], examined the association between sub-chronic exposure (in between acute and chronic) to six outdoor air pollutants, PM_2.5_, PM_10_, O_3_, NO_2_, SO_2_, CO, with both hospital costs and length of stay of pediatric asthma hospitalization in the U.S. The study found that a 1-unit (μg/m^3^) increase in monthly PM_2.5_ led to a $123 increase in charges (95% CI: $40–249) and a $47 increase in costs (95% CI: $15–93). This study only looked at two cost factors, hospital costs and length of stay of pediatric asthma hospitalization in the U.S., while our study looked at both direct and indirect costs. 

Our study, using the estimates from Nurmagambetov et al. [21], put a total cost of a pediatric asthma case on average to be $1196 in the U.S. Our study’s direct costs were the medical cost to a payer. A payer’s medical costs is the sum of all payments for emergency room visits, office-based medical provider visits, outpatient hospital visits, and prescription medications. As they used the MEPS dataset, the medical costs are payer’s expenses and represent the total annual amount that a payer spent on their medical care and prescription medications.

### 4.2. Strengths and Limitations

Our results are all estimates based on the best available and peer reviewed literature, but they are still estimates with many limitations, as explained below. We provide a 95% CI around each state’s central estimate to provide a range of uncertainty. The results of our estimation are subject to several limitations. Any limitations from our two source studies [16,21] will carry over to this study. Khreis et al. [16] selected NO_2_ as the exposure of interest (thus so did we), as it has been the most commonly used pollutant in previous BoD analyses and a larger body of studies supports its use as an appropriate CRF [9]. NO_2_ is also a relatively good marker for TRAP, compared to PM_2.5_ and PM_10_, which are the two other pollutants for which we have childhood asthma BoD estimates [15]. It can be argued, however, that NO_2_ is not the putative agent but rather serves as a surrogate pollutant for the mixture of fresh traffic exhaust and its variation in urban areas. There is stronger and more toxicological evidence that links particulate matter with incident asthma and emerging evidence to suggest that certain chemical components of particulate matter, such as black carbon, ammonium, and nitrates, may be more relevant in the onset of asthma [13,42]. However, this issue was less relevant in the original analysis by Khreis et al. [16], as the only aim of that study was to establish the potential impact of using different baseline asthma incidence rates on the final burden of disease estimates. Possible interactions between pollutants were not considered, as there are very limited epidemiological data to investigate this, and as such, we do not comment on this matter further. Khreis et al. [16] also had limitations in the datasets they used to estimate the state-specific asthma incidence rates, and these limitations are relevant here. The total childhood samples included for the period 2006–2010 were 293,464 samples from the BRFSS and 16,156 samples from the ACBS. These samples were, however, weighted to represent the total number of children within each state, with similar characteristics (e.g., age, sex, and race). In other words, weights were used to convert samples to population estimates of children. A larger sample size in these surveys may have more accurately represented the U.S. childhood population of approximately 73 million in 2010, which we included in our analysis, but this is not established. Furthermore, NO_2_ exposures were estimated for the year 2010, whereas the estimation of the asthma incidence rates utilized data from 2006 to 2010. The reason why Khreis et al. [16] included earlier years of data in the estimation of their asthma incidence rates was because many states did not have survey results for the year 2010, and relying on 2010 data only would mean that they would have to exclude those states from the analysis, and therefore, not cover the whole of the U.S. Importantly, because the estimation of asthma incidence rates was carried out at a finer spatial resolution in their study (at the state versus the national level), the sample sizes available for their estimation were reduced, and using the aggregate data for the years 2006 to 2010 increases the sample sizes and, therefore, the confidence in the incidence rate estimates. Ideally, enough data would have been available to allow a complete and robust calculation of asthma incidence rates for the year 2010 specifically, but as the aim of their study was to establish the potential impact of using different incidence rates only, this aim was not compromised using aggregate survey data over the years 2006 to 2010. Khreis et al. [16] relied on state-specific pediatric asthma incidence rates in their analysis, but sub-state variations most likely exist, including between urban and rural populations, and across different races, sexes, ages, and socioeconomic status [16]. Unfortunately, this information was not readily available, limiting the ability to estimate subpopulation specific asthma incidence rates within each state; however, this is also less relevant to the aim of this study, which is to conduct an economic analysis showing the total cost of childhood asthma incident cases attributable to NO_2_ in each state.

The LUR models used by Khreis et al. [16] estimated concentrations at the centroid of census blocks, which could be a farther point from roadways since census blocks are usually classified by roadways. They were unable to verify how this would affect the direction of exposure or exposure misclassification since calculating the average concentration at a finer scale within census blocks was not feasible due to the large computational intensity needed to predict values across the whole of the U.S. [15,16,29].

The MEPS dataset was used as the primary source of data by Nurmagambetov et al. [21]. This dataset may underestimate the total medical costs, because first, it does not capture all medical care consumption and costs. Some of the use that was not captured may have included non-durable goods, such as over-the counter medications or prescription drugs, and health services administered in non-medical settings, such as health screenings delivered in non-health establishments such as the workplace and school. Second, a comparison of the MEPS data with other medical use data revealed that MEPS respondents tend to underreport medical events, which can lead to an underestimate of use and thus costs [43,44,45].

The estimates from Nurmagambetov et al. [21] of the per person costs are based on a national regression model. Even though they applied state level adjustment factors from the 2003 restricted used MEPS data, their results are still based on an assumption that the geographical difference in medical costs across the states has remained the same since 2003. This is relevant as we were not able to have the most up to date and accurate medical costs according to each state for the year of analysis. 

Nurmagambetov et al. [21] also cautioned against a direct comparison of the costs across states for two reasons. First, the costs were derived from data across different levels of geographic levels. Second, survey weights from MEPS were not intended to be representative of state populations. This is pertinent as using MEPS may not have accurately characterized state populations and costs. Additionally, while the authors provided absenteeism cost of asthma as an important measure of indirect costs to the society, there were additional indirect cost factor that were not considered. These include estimates of productivity losses through caregiving to adults with asthma, household productivity losses, reduced productivity while at work, drops in the quality of life, and premature mortality. This may have caused a lower estimate of indirect costs when comparing with studies such as Brandt et al. [25], where the value of the caregiver’s time spent traveling, waiting, and receiving care were included in addition to missed workdays. In addition to the above limitations from our two source studies, our study has other limitations. 

First, our two source studies, Nurmagambetov et al. [21] and Khreis et al. [16], defined asthma differently. Nurmagambetov et al. [21] defined childhood asthma as it was defined in MEPS. In MEPS, self-reported conditions are recorded by professional coders using ICD-9-CM codes and are then grouped into clinical classification system (CCS) codes. Nurmagambetov et al. [21] defined asthma using ICD-9-CM code 493 and CCS code 128 and used only cases associated with reported medical use. Specifically, a MEPS respondent with asthma ICD-9-CM or CCS codes was only considered to have a case of medically treated asthma if they had at least one of the following medical procedures: a hospitalization, an emergency department visit, an office-based medical provider visit, an outpatient hospital visit, or the reported use of prescription drugs. Nurmagambetov et al. [21] categorized people with no asthma-related medical events during that year as not having medically treated asthma, regardless of if the person had a previous diagnosis of asthma. This exclusion could underestimate the future cost of asthma for those with the condition, as it does not account for possible future relapses. 

On the other hand, Khreis et al. [16] determined the asthma status of children through the BRFSS and the nested ACBS. To determine the asthma status of children, respondents to the BRFSS were asked “Has a doctor, nurse, or other health professional ever said that the child has asthma?” If the answer was “yes,” the respondent was designated as “ever asthma.” If the answer was “no,” the respondent was designated as “never asthma.” Respondents with children designated as “ever asthma” were requested to participate in the ACBS follow-up. To determine the “incident status” of children, respondents to the ACBS were asked “How old was [name of child] when a doctor or other health professional first said [he/she] had asthma? How long ago was that?” If the answer to the latter part of this question was “within the past 12 months,” the respondent was designated as an “incident asthma.” Both studies, since they used different asthma definitions, could be capturing different asthma populations and different asthma severities.

Another limitation in this study is presenting the costs as a total for all the U.S. by adding all the states’ total costs together. When looking at these numbers, it is important to note that Nurmagambetov et al. [21] do not total their cost numbers. As mentioned above, they caution against a direct comparison of the results across states.

Our study’s strengths include being one of the first to provide a comprehensive assessment of the economic cost of childhood asthma cases attributable to NO_2_ across the contiguous U.S. Furthermore, throughout the research process, we discovered a lack of research on the burden of pediatric asthma costs. Some comprehensive studies did report the childhood cost of asthma but did not provide a breakdown of the burden by direct and indirect costs [23]. Moreover, no study has used this information yet to estimate the economic burden of childhood asthma attributable to TRAP. This study fills this gap, although our estimates are subject to some limitations (outlined above) and may represent an underestimation of the true cost of the disease, as already discussed. 

### 4.3. Policy Implications

Air pollution continues to be a global threat to human health, contributing to a substantial but modifiable BoD and premature mortality. About 90% of people breathe air that does not comply with the World Health Organization Air Quality Guidelines [46]; guidelines which are still too high to protect human health [16]. According to a 2015 global BoD Study, exposure to PM_2.5_ is the fifth leading risk factor for death worldwide, accounting for 4.2 million deaths and 103.1 million disability-adjusted life-years in 2015 [47]. Air pollution also has several effects on different health issues including, but not limited to, cardiovascular disease, lung cancer, diabetes, adverse birth outcomes, congenital anomalies, pregnancy-induced hypertensive disorders and preeclampsia, and adverse respiratory outcomes beyond asthma, especially in susceptible populations such as children and the elderly. The list of associated health effects continues to grow and now includes health effects which were not associated with TRAP a decade or so ago, such as autism and child behavioral problems, cognitive decline, dementia and Alzheimer’s disease, obesity, and an increased number of osteoporosis-related fracture hospital visits and decreased bone density [48]. Adverse health effects associated with TRAP continue to emerge at a very rapid pace [49], and the body of evidence has been strengthened substantially to demand urgent action [48,50,51,52]. 

The economic burden of air pollution is also high. Health-related costs from the effects of O_3_ air pollution exceeding national standards have been estimated at $6.5 billion nationwide (in 2008 U.S. dollars), based on a U.S. assessment of health impacts from O_3_ levels during 2000–2002 [53]. In the UK, the associated annual health costs of health outcomes resulting from exposure to air pollution have been estimated at between £22.6 billion and £71.3 billion [54]. Data generated by the Centre for Research on Energy and Clean Air and Greenpeace model suggests that an estimated global annual cost of U.S. $2.9 trillion (central estimate), equivalent to 3.3% of global Gross Domestic Product (GDP) or U.S. $8 billion per day, is attributed to air pollution from fossil fuels. Costs of U.S. $350 billion and U.S. $380 billion are estimated to be attributed to NO_2_ and O_3_ air pollution from fossil fuels, respectively, each equivalent to 0.4% of the global GDP [55].

While costing exercises have been mainly conducted for mortality impacts in the past, assessing the cost of morbidity endpoints is also important and encouraged, especially when the disease in question is chronic and carries over the life course of individuals. One of these endpoints is childhood asthma. We think it is important to understand the cost of TRAP-related childhood asthma incidence due to its economic implications and the fact that this disease is chronic. When chronic bronchitis (a form of COPD) had been included in EPA risk assessments in the past, this component of the health impact monetary valuation was the largest morbidity effect, exceeded only by mortality in dollar valuation, showing the importance of including the cost of morbidity. This high cost is because, a person getting a chronic disease is a long-term adverse effect on their quality of life and, therefore, there could be a high willingness to pay-to-avoid dollar value associated with new onset of chronic diseases.

If we reduce TRAP, we can reduce childhood asthma cases, acute and chronic, and reduce the financial burden of it. With a reduced financial burden, there would be increased Medicare and Medicaid budgets in states that could be used for addressing other health issues. Estimates that assume no change in regulatory controls or population characteristics have ranged from 1000 to 4300 additional premature deaths nationally per year by 2050 from combined O_3_ and particle health effects according to the Centers of Disease Control [54]. As the first study to monetize the cost of childhood asthma attributable to NO_2_ in the U.S., we urge for future research on the topic to reaffirm our findings and put them in perspective, in addition to starting to investigate the costs of other chronic endpoints which have been convincingly associated with air pollution.

These estimates should be factored into the regulatory framework for air quality standards and in cost-benefits analyses of projects with an air pollution impact, including transportation infrastructure and planning projects. Policy makers could also respond in a few other ways, first by increasing funding for studies related to air pollution and health research, especially the monetization of these impacts, and second, by focusing on strategies on how to mitigate TRAP and human exposures. 

## 5. Conclusions

This study conducted the first state level economic analyses on TRAP-attributable childhood asthma cases in the U.S. to help provide information on the high costs of TRAP-attributable health impacts. The results of the analysis suggest that the total burden in the states to be $178,900,138.989 (95% CI: $101,019,728.20–$256,980,126.65), which is likely underestimated, as we showed in our discussion. Our analysis suggests $178,900,138.989 is 19.8% of all asthma costs. This demonstrates that TRAP, represented by NO_2_ in this work, has large economic and health impacts. Future estimates need to capture the most accurate pediatric asthma case count across the U.S., in addition to the most accurate cost of treating asthma, as costs can vary, and any additional health problems that may be exacerbated due to asthma, as well as their costs, to get a full and more accurate picture of the cost burden of pediatric asthma. 

The present-day cost of asthma estimates based on nationally recognized databases and thorough econometric analysis are vital, as they can assist state policymakers, program managers, and evaluators in reviewing options to reduce the economic asthma burden in their state. A literature review performed on existing methodologies to estimate air pollution-related health impacts and subsequent external costs found that although many studies try to analyze the economic impact of air pollution, most end up omitting some measurable cost components and have limitations with respect to the methods used in estimating damage costs. We also highlight a need for increased public access to healthcare spending data in the U.S. to encourage more research on this topic, which was an issue we came across in our study. 

The high economic cost shows the increased financial burden of air pollution and its effects on American families and the state and national health care systems. Previous studies have already shown the need to focus on reducing TRAP in the U.S. [15,16], a conclusion that is further backed by this study’s results. With the high percentage of pediatric asthma costs attributable to NO_2_, we are clearly highlighting how TRAP is having a direct impact both financially and medically on children with asthma, their families, the healthcare system, and its ancillaries, such as insurance companies in the U.S.

## Figures and Tables

**Table 1:** Summary of previous studies.

Previous Review	Previous Review Scope	Pollutants Studied	Region Studied	Population	Years Studied	Key Differences to Our Study
[26]	Estimated outdoor air pollution’s short-term effect on daily asthma-related patient visits and their economic costs. Calculated economic cost of patient visits by pollutant.	SO_2_, CO, NO_2_, PM_10_, O_3_, and PM_2.5_	Shanghai, China	The patient pool was from a local hospital. Age was not specified.	2014–2015	Scope focuses only on the short-term effect of air pollution. Limited region study to Shanghai, China. Patient age not specified to specific group. Only analyzed the cost variable of asthma patient visits.
[25]	Estimates the cost of childhood asthma caused by near roadway pollution (NRP). Calculated the direct cost of medical goods and services, and indirect costs of caregivers’ lost wages, as well as willingness to pay.	NRP, regional O_3_, and NO_2_	Los Angeles County, California	Focused on the effect on childhood asthma cases, aged 0–17.	2007	Studied hypothetical scenarios involving a decrease in pollution and a decrease or increase in population density within 75 m of a major roadway. Limited region study to Los Angeles County. Analyzed the cost effect of the willingness to pay and only looked at the indirect cost of the caregiver and not the child. Analyzed NRP, NO_2_, and O_3_ exposures.
[28]	Estimates the cost of childhood asthma cases attributable to air pollution. Calculated the direct and indirect cost and the cost of bronchitis episodes in addition to asthma cost. Costs are in 2010 dollars.	NO_2_ and O_3_	Riverside and Long Beach, California.	Focused on the effect on childhood asthma cases, aged 0–17.	N/A	Studied NO_2_ and O_3_. Limited region to two California cities. Looked specifically at the unique economic effect of bronchitis episodes had on costs in addition to asthma. Study is not specified to a year.
[27]	Examines the association between sub-chronic (the authors defined the following exposures: an exposure duration of less than 14 days is acute, more than 14 days up to one year is sub-chronic, and greater than one year is chronic) exposure (in between acute and chronic) to six outdoor air pollutants with both costs and length of stay of pediatric asthma hospitalization in the U.S.	PM_2.5_, PM_10_, O_3_, NO_2_, SO_2_, CO	U.S.	Studies the effects on pediatric asthma, aged 12–17.	1999–2007	Scope focuses only on direct costs of pediatric asthma hospitalizations. Studied PM_2.5_, PM_10_, O_3_, NO_2_, SO_2_, and CO.

**Table 2:** Database review.

Database	Year of Data	Direct Costs	Indirect Costs	Childhood Costs	State (U.S.) Focused	National (U.S.) Focused
HCUPnet	2000	✓		✓		✓
HCUPnet	2010	✓		✓		✓
HCUPnet	2000	✓		✓	✓	
HCUPnet	2010	✓		✓	✓	
HCUPnet	2010	✓		✓		✓
AHRQ MEPS Summary Tables	2000	✓		✓		✓
AHRQ MEPS Summary Tables	2010	✓		✓		✓

Note: HCUPnet is an online query system based on data from the Healthcare Cost and Utilization Project (HCUP). The Agency for Healthcare Research and Quality (AHRQ) Medical Expenditure Panel Survey (MEPS) summary tables provide frequently used summary estimates for the U.S. civilian population on household medical utilization and expenditures, demographic and socio-economic characteristics, health insurance coverage, access to care and experience with care, medical conditions, and prescribed medicine purchases.

**Table 3:** Published studies review.

Database	Direct Costs	Indirect Costs	Childhood Costs	State (U.S.) Focused	National (U.S.) Focused
Barrett et al. [33]	✓		✓		✓
Barnett and Nurmagambetov. [34]	✓	✓	✓		✓
Wang et al. [35]	✓	✓			✓
Nurmagambetov et al. [21]	✓	✓	✓	✓	
Nurmagambetov et al. [23]	✓	✓	✓		✓
Sullivan et al. [36]	✓		✓		✓
Karaca-Mandic et al. [37]	✓		✓		✓

**Table 4:** State annual economic analysis.

State	Number of Childhood Asthma Incident Cases Attributable to NO_2_ (95% CI)	Per Person Direct Medical Costs	Total Direct Medical Costs of Childhood Asthma Incident Cases Attributable to NO_2_ (95% CI)	Per Person Indirect Medical Costs	Total Indirect Cost of Childhood Asthma Incident Cases Attributable to NO_2_ (95% CI)	Total Indirect and Direct Costs of Childhood Asthma Incident Cases Attributable to NO_2_ (95% CI)
Alabama	1330 (1330–1330)	$983	$1,307,390 ($1,307,390–$1,307,390)	$309.02	$410,998.08 ($410,998.08–$410,998.08)	$1,718,388.08 ($1,718,388.08–$1,718,388.08)
Arizona	4623 (0–9273)	$833	$3,850,959 ($0–$7,724,409)	$337.35	$1,559,566.27 ($0.00–$3,128,240.96)	$5,410,525.27 ($0.00–$10,852,649.96)
Arkansas	945 (945–945)	$860	$812,700 ($812,700–$812,700)	$287.88	$272,045.45 ($272,045.45–$272,045.45)	$1,084,745.45 ($1,084,745.45–$1,084,745.45)
California	19205 (7854–30,555)	$864	$16,593,120 ($6,785,856–$26,399,520)	$411.81	$7,908,739.84 ($3,234,326.62–$12,582,741.25)	$24,501,859.84 ($10,020,182.62–$38,982,261.25)
Colorado	3292 (3291–3292)	$1076	$3,542,192 ($3,541,116–$3,542,192)	$383.60	$1,262,804.23 ($1,262,420.63–$1,262,804.23)	$4,804,996.23 ($4,803,536.63–$4,804,996.23)
Connecticut	1502 (818–2186)	$1084	$1,628,168 ($886,712–$2,369,624)	$416.33	$625,334.66 ($340,561.75–$910,107.57)	$2,253,502.66 ($1,227,273.75–$3,279,731.57)
Delaware	386 (26–746)	$1032	$398,352 ($26,832–$769,872)	$378.95	$146,273.68 ($9,852.63–$282,694.74)	$544,625.68 ($36,684.63–$1,052,566.74)
D.C.	313 (313–314)	$1023	$320,199 ($320,199–$321,222)	$612.24	$191,632.65 ($191,632.65–$192,244.90)	$511,831.65 ($511,831.65–$513,466.90)
Florida	5863 (5862–5864)	$976	$5,722,288 ($5,721,312–$5,723,264)	$319.33	$1,872,245.02 ($1,871,925.69–$1,872,564.35)	$7,594,533.02 ($7,593,237.69–$7,595,828.35)
Georgia	2772 (1353–4190)	$935	$2,591,820 ($1,265,055–$3,917,650)	$340.54	$943,965.43 ($460,745.03–$1,426,845.29)	$3,535,785.43 ($1,725,800.03–$5,344,495.29)
Idaho	571 (571–571)	$993	$567,003 (567,003–$567,003)	$305.34	$174,351.15 ($174,351.15–$174,351.15)	$741,354.15 ($741,354.15–$741,354.15)
Illinois	4509 (186–8832)	$1013	$4,567,617 ($188,418–$8,946,816)	$375.35	$1,692,453.78 ($69,815.13–$3,315,092.44)	$6,260,070.78 ($258,233.13–$12,261,908.44)
Indiana	3852 (2450–5254)	$1030	$3,967,560 ($2,523,500–$5,411,620)	$314.44	$1,211,222.67 ($770,377.87–$1,652,067.48)	$5,178,782.67 ($3,293,877.87–$7,063,687.48)
Iowa	519 (260–777)	$984	$510,696 ($255,840–$764,568)	$309.52	$160,642.86 ($80,476.19–$240,500.00)	$671,338.86 ($336,316.19–$1,005,068.00)
Kansas	787 (533–1040)	$973	$765,751 ($518,609–$1,011,920)	$319.40	$251,370.15 ($170,241.79–$332,179.10)	$1,017,121.15 ($688,850.79–$1,344,099.10)
Kentucky	1532 (1532–1532)	$984	$1,507,488 ($1,507,488–$1,507,488)	$302.97	$464,144.07 ($464,144.07–$464,144.07)	$1,971,632.07 ($1,971,632.07–$1,971,632.07)
Louisiana	653 (0–1449)	$895	$584,435 ($0–$1,296,855)	$303.68	$198,299.81 ($0.00–$440,025.15)	$782,734.81 ($0.00–$1,736,880.15)
Maine	173 (69–277)	$933	$161,409 ($64,377–$258,441)	$321.43	$55,607.14 ($22,178.57–$89,035.71)	$217,016.14 ($86,555.57–$347,476.71)
Maryland	2454 (1501–3406)	$929	$2,279,766 ($1,394,429–$3,164,174)	$409.97	$1,006,061.09 ($615,361.74–$1,396,350.48)	$3,285,827.09 ($2,009,790.74–$4,560,524.48)
Massachusetts	2705 (2705–2706)	$997	$2,696,885 ($2,696,885–$2,697,882)	$434.88	$1,176,353.34 ($1,176,353.34–$1,176,788.22)	$3,873,238.34 ($3,873,238.34–$3,874,670.22)
Michigan	4056 (2554–2558)	$1121	$4,546,776 ($2,863,034–$6,230,518)	$340.55	$1,381,253.06 ($869,753.53–$1,892,752.59)	$5,928,029.06 ($3,732,787.53–$8,123,270.59)
Minnesota	2045 (2045–2046)	$999	$2,042,955 ($2,042,955–$2,043,954)	$370.99	$758,684.65 ($758,684.65–$759,055.65)	$2,801,639.65 ($2,801,639.65–$2,803,009.65)
Mississippi	929 (275–1583)	$860	$798,940 ($236,500–$1,361,380)	$274.57	$255,072.25 ($75,505.78–$434,638.73)	$1,054,012.25 ($312,005.78–$1,796,018.73)
Missouri	1898 (443–3353)	$907	$1,721,486 ($401,801–$3,041,171)	$326.19	$619,102.60 ($144,500.77–$1,093,704.44)	$2,340,588.60 ($546,301.77–$4,134,875.44)
Montana	69 (26–112)	$994	$68,586 ($25,844–$111,328)	$294.12	$20,294.12 ($7,647.06–$32,941.18)	$88,880.12 ($33,491.06–$144,269.18)
Nebraska	494 (298–690)	$972	$480,168 ($289,656–$670,680)	$313.08	$154,663.55 ($93,299.07–$216,028.04)	$634,831.55 ($382,955.07–$886,708.04)
Nevada	1377 (1377–1377)	$994	$1,368,738 ($1,368,738–$1,368,738)	$330.05	$454,477.83 ($454,477.83–$454,477.83)	$1,823,215.83 ($1,823,215.83–$1,823,215.83)
New Hampshire	329 (163–496)	$935	$307,615 ($152,405–$463,760)	$352.60	$116,005.78 ($57,473.99–$174,890.17)	$423,620.78 ($209,878.99–$638,650.17)
New Jersey	4155 (2321–5989)	$1034	$4,296,270 ($2,399,914–$6,192,626)	$415.17	$1,725,023.46 ($963,605.16–$2,486,441.75)	$6,021,293.46 ($3,363,519.16–$8,679,067.75)
New Mexico	471 (215–726)	$993	$467,703 ($213,495–$720,918)	$318.47	$150,000.00 ($68,471.34–$231,210.19)	$617,703.00 ($281,966.34–$952,128.19)
New York	13,504 (7076–10,932)	$1003	$13,544,512 ($7,097,228–$19,991,796)	$424.25	$5,729,122.39 ($3,002,019.40–$8,456,225.37)	$19,273,634.39 ($10,099,247.40–$28,448,021.37)
North Carolina	3390 (3390–3391)	$976	$3,308,640 ($3,308,640–$3,309,616)	$329.26	$1,116,180.62 ($1,116,180.62–$1,116,509.88)	$4,424,820.62 ($4,424,820.62–$4,426,125.88)
North Dakota	137 (137–137)	$971	$133,027 ($133,027–$133,027)	$323.94	$44,380.28 ($44,380.28–$44,380.28)	$177,407.28 ($177,407.28–$177,407.28)
Ohio	6165 (3011–9319)	$927	$5,714,955 ($2,791,197–$8,638,713)	$334.94	$2,064,903.61 ($1,008,503.61–$3,121,303.61)	$7,779,858.61 ($3,799,700.61–$11,760,016.61)
Oklahoma	1154 (620–1687)	$944	$1,089,376 ($585,280–$1,592,528)	$308.76	$356,304.15 ($191,428.57–$520,870.97)	$1,445,680.15 ($776,708.57–$2,113,398.97)
Oregon	1180 (282–2078)	$997	$1,176,460 ($281,154–$2,071,766)	$359.85	$424,621.21 ($101,477.27–$747,765.15)	$1,601,081.21 ($382,631.27–$2,819,531.15)
Pennsylvania	6305 (1764–10,846)	$980	$6,178,900 ($1,728,720–$10,629,080)	$345.89	$2,180,814.02 ($610,143.68–$3,751,484.36)	$8,359,714.02 ($2,338,863.68–$14,380,564.36)
Rhode Island	422 (130–713)	$933	$393,726 ($121,290–$665,229)	$372.26	$157,094.89 ($48,394.16–$265,423.36)	$550,820.89 ($169,684.16–$930,652.36)
South Carolina	1371 (1371–1372)	$961	$1,317,531 ($1,317,531–$1,318,492)	$300.00	$411,300.00 ($411,300.00–$411,600.00)	$1,728,831.00 ($1,728,831.00–$1,730,092.00)
South Dakota	176 (176–176)	$972	$171,072 ($171,072–$171,072)	$287.23	$50,553.19 ($50,553.19–$50,553.19)	$221,625.19 ($221,625.19–$221,625.19)
Tennessee	2667 (2667–2667)	$1096	$2,923,032 ($2,923,032–$2,923,032)	$308.36	$822,389.05 ($822,389.05–$822,389.05)	$3,745,421.05 ($3,745,421.05–$3,745,421.05)
Texas	14,316 (7776–20,856)	$961	$13,757,676 ($7,472,736–$20,042,616)	$345.38	$4,944,481.93 ($2,685,686.75–$7,203,277.11)	$18,702,157.93 ($10,158,422.75–$27,245,893.11)
Utah	1672 (1048–2295)	$991	$1,656,952 ($1,038,568–$2,274,345)	$325.93	$544,948.15 ($341,570.37–$748,000.00)	$2,201,900.15 ($1,380,138.37–$3,022,345.00)
Vermont	126 (81–1710	$934	$117,684 ($75,654–$159,714)	$333.33	$42,000.00 ($27,000.00–$57,000.00)	$159,684.00 ($102,654.00–$216,714.00)
Virginia	3200 (3200–3201)	$1011	$3,235,200 ($3,235,200–$3,236,211)	$387.21	$1,239,069.77 ($1,239,069.77–$1,239,456.98)	$4,474,269.77 ($4,474,269.77–$4,475,667.98)
Washington	1703 (950–2456)	$995	$1,694,485 ($945,250–$2,443,720)	$404.12	$688,222.68 ($383,917.53–$992,527.84)	$2,382,707.68 ($1,329,167.53–$3,436,247.84)
West Virginia	578 (42–1114)	$1033	$597,074 ($43,386–$1,150,762)	$286.52	$165,606.74 ($12,033.71–$319,179.78)	$762,680.74($55,419.71–$1,469,941.78)
Wisconsin	2154 (1–4307)	$1081	$2,328,474 ($1,081–$4,655,867)	$325.73	$701,628.66 ($325.73–$1,402,931.60)	$3,030,102.66($1,406.73–$6,058,798.60)
Wyoming	138 (138–138)	$992	$136,896 ($136,896–$136,896)	$341.46	$47,121.95 ($47,121.95–$47,121.95)	$184,017.95($184,017.95–$184,017.95)
Total:			**$129,950,707 ($73,785,005–$186,264,165)**		**$48,949,431.98 ($27,234,723.20** **–** **$70,715,961.65)**	**$178,900,138.989 ($101,019,728.20–$256,980,126.65)**

Note: All costs are in 2014 dollars.

## Data Availability

The number of childhood asthma incident cases attributable to NO_2_ and the 95% CI were retrieved from Table S5 from the Khreis et al. [16] study [https://www.sciencedirect.com/science/article/pii/S1047279720303446?via%3Dihub, accessed on 19 September 2020]. We retrieved both per person direct and indirect costs from Table 2 and Table 4 respectively from the Nurmagambetov et al. [21] [https://www-tandfonline-com.srv-proxy2.library.tamu.edu/doi/full/10.1080/02770903.2016.1218013, accessed on 25 May 2021].

## Supplementary Materials

The following are available online at https://www.mdpi.com/article/10.3390/ijerph18157864/s1, Table S1: Database Review, Table S2: Literature Review, Table S3: Cost Calculations.

## Abbreviations

ACBS	Asthma Call-Back Survey
AHRQ	Agency for Healthcare Research and Quality
BLS	Bureau of Labor Statistics
BoD	Burden of Disease
BRFSS	Behavioral Risk Factor Surveillance System
CCS	Clinical Classification System
CI	Confidence Interval
CO	Carbon Monoxide
COB	Congressional Budget Office
CRF	Concentration Response Function
D.C.	Washington D.C.
GDP	Gross Domestic Product
ICD-9-CM	International Classification of Diseases, Ninth Revision, Clinical Modification
LUR	Land-Use Regression
HCUP	Health Care Cost and Utilization Project
MEPS	Medical Expenditure Panel Survey
NRP	Near Roadway Pollution
NO_2_	Nitrogen Dioxide
NOx	Nitrogen Oxides
O_3_	Ozone
PAF	Population-Attributable Fraction
PM_10_	Particulate Matter with a Diameter less than 10 micrometers
PM_2.5_	Particulate Matter with a Diameter less than 2.5 micrometers
TRAP	Traffic-Related Air Pollution
U.S.	United States
WTP	Willingness to Pay
