# Supplementary material for: Monetizing the Burden of Childhood Asthma Due to Traffic Related Air Pollution in the Contiguous United States in 2010
Source: Int J Environ Res Public Health. 2021 Jul 25;18(15):7864. doi: 10.3390/ijerph18157864 (PMC8345553; doi:10.3390/ijerph18157864)
Supplement: Supplementary file 1 [file ijerph-18-07864-s001.zip › Supplementary Tables.pdf]

ABBREVIATIONS for Supplementary Materials

493.0	Extrinsic asthma with status asthmaticus
493.02	Extrinsic asthma with (acute) exacerbation
493.10	Intrinsic asthma, unspecified
493.11	Intrinsic asthma with status asthmaticus
493.12	Intrinsic asthma with (acute) exacerbation
493.20	Chronic obstructive asthma, unspecified
493.21	Chronic obstructive asthma with status asthmaticus
493.22	Chronic obstructive asthma with (acute) exacerbation
493.90	Asthma, unspecified type, unspecified
493.91	Asthma, unspecified type, with status asthmaticus
493.92	Asthma, unspecified type, with (acute) exacerbation
493	Asthma
493.0	Extrinsic Asthma,
493.1	Intrinsic Asthma
493.2	Chronic Obstructive Asthma
493.9	Asthma Unspecified
493.8	Other forms of asthma
493.81	Exercise induced bronchospasm
493.82	Cough variant asthma
HCUP	Healthcare Cost and Utilization Project
ICD-9-CM	International Classification of Diseases, Ninth Revision, Clinical Modification
KID	Kid’s Inpatient Database
MEPS	Medical Expenditure Survey
NIS	National Inpatient Sample
ED	Emergency Department

Table S1: Database Review

Reference	Data Source, Data Type, Location	Direct Costs	Indirect Costs	Sample Size	Age	Year	How is Asthma Defined	How are Costs Defined?
**HCUPnet, Healthcare Cost and Utilization Project. Agency for Healthcare Research and Quality, Rockville, MD.**	HCUPnet, Inpatient, National (United States (U.S.))	The mean charge per discharge is $5,667.	N/A	The sample for the cost estimates in this study used the Healthcare Cost and Utilization (HCUP) Kid's Inpatient Database (KID). The total number of weighted discharges in the U.S. based on the HCUP KID is 6,351,345. The direct cost for the mean charge per discharge is calculated from 123,054 discharges.	0-17.	2000	Asthma is defined by the International Classification of Diseases, Ninth Revision, Clinical Modification (ICD-9-CM) diagnoses codes: 493.00, 493.01,493.02, 493.10,493.11, 493.12, 493.20, 493.21, 493.22, 493.90, 493.91, 493.92, 493, 493.0,493.1, 493.2, 493.9.	• The data sets from the HCUP provides both costs and charges in their analysis.<br>• Charges are represented in the direct cost column.<br>• Costs reflect the actual costs of production, whereas charges represent what the hospital billed for the stay.<br>• The costs and charges represented are by each discharge and not by each person. If or when calculating costs, the rate of inpatient stays per U.S. children needs to be established to allow the calculation of costs per child**.**
**HCUPnet, Healthcare Cost and Utilization Project. Agency for Healthcare Research and Quality, Rockville, MD.**	HCUPnet, Inpatient, National (U.S.)	For Ages <1: The mean charge per discharge is $11,135.<br>For Ages 1-17: The mean charge per discharge is $11,552.	N/A	The sample for the cost estimates is from the HCUP National Inpatient Sample (NIS). The total number of weighted discharges in the U.S. based on the HCUP NIS is 37,352,013.<br>For Ages <1, the mean charge per discharge calculation is based on 7,137 discharges. For Ages 1-17, the mean charge per discharge calculation is based on 110,602 discharges.	<1, 1-17	2010	Asthma is defined by ICD-9-CM diagnoses codes: 493.00, 493.01,493.02, 493.10,493.11, 493.12, 493.20, 493.21, 493.22, 493.90, 493.91, 493.92, 493, 493.0,493.1, 493.2, 493.9, 493.8, 493.81, 493.82	• The data sets from the HCUP provides both costs and charges in their analysis.<br>• Charges are represented in the direct cost column.<br>• Costs reflect the actual costs of production, whereas charges represent what the hospital billed for the stay.<br>• The costs and charges represented are by each discharge and not by each person. If or when calculating costs, the rate of inpatient stays per U.S. children needs to be established to allow the calculation of costs per child.<br>• The HCUP KID, the children only database, was only available for 2000. The costs presented are from the HCUP NIS database. This database divides the under 18 age groups in two, <1, 1-17.

**HCUPnet, Healthcare Cost and Utilization Project. Agency for Healthcare Research and Quality, Rockville, MD.**	HCUPnet, Inpatient, U.S. States	The total mean charge per discharge for the age group under 1 ranged from $2,825 to $8,719.<br>The total charge per discharge for the age group between 1 and 17 ranged from $3,446 to $9,165.	N/A	These estimates are from the HCUP NIS database. Sample numbers vary by each state.<br><br>The discharge numbers for the <1 age group used to calculate costs ranged from 50 to 1675; for the 1-17 age group, it ranges from 364 to 14,411. The average number of discharges of for the <1 age group is about 451 and for the 1-17 age group is 3793.	<1, 1-17	2000	Asthma is defined by ICD-9-CM diagnoses codes:<br>493.00, 493.01,493.02, 493.10,493.11, 493.12, 493.20, 493.21, 493.22, 493.90, 493.91, 493.92, 493, 493.0,493.1, 493.2, 493.9, 493.8, 493.81, 493.82	• The data sets from the HCUP provides both costs and charges in their analysis.<br>• Charges are represented in the direct cost column.<br>• Costs reflect the actual costs of production, whereas charges represent what the hospital billed for the stay.<br>• The costs and charges represented are by each discharge and not by each person. If or when calculating costs, the rate of inpatient stays per U.S. children needs to be established to allow the calculation of costs per child.<br>• The HCUP KID, the children only database, was not available for 2000. The costs presented are from the HCUP NIS database. This database divides the under 18 age groups in two, <1, 1-17.<br>• Costs were not available from all states.
**HCUPnet, Healthcare Cost and Utilization Project. Agency for Healthcare Research and Quality, Rockville, MD.**	HCUPnet, Inpatient, U.S. States	The total mean charge per discharge for the age group under 1 ranged from $4388 to $17,820.75.<br>The total charge per discharge for the age group between 1 and 17 ranged from $5319.04 to $22,605.	N/A	These estimates are from the HCUP National (Nationwide Inpatient Sample (NIS). Sample numbers vary by each state. The Direct Costs column shows the number of discharges the mean costs were calculated from. The discharge numbers for the <1 age group used to calculate costs ranged from 12 to 822; for the 1-17 age group, it ranges from 53 to 11147. The average number of discharges of for the <1 age group is about 176 and for the 1-17 age group is 2450.	<1, 1-17	2010	ICD-9-CM Diagnoses Codes:<br>493.00, 493.01,493.02, 493.10,493.11, 493.12, 493.20, 493.21, 493.22, 493.90, 493.91, 493.92, 493, 493.0,493.1, 493.2, 493.9, 493.8, 493.81, 493.82	• The data sets from the HCUP provides both costs and charges in their analysis.<br>• Charges are represented in the direct cost column.<br>• Costs reflect the actual costs of production, whereas charges represent what the hospital billed for the stay.<br>• The costs and charges represented are by each discharge and not by each person. If or when calculating costs, the rate of inpatient stays per U.S. children needs to be established to allow the calculation of costs per child.<br>• The HCUP KID, the children only database, was not available for 2010. The costs presented are from the HCUP NIS database. This database divides the under 18 age groups in two, <1, 1-17.<br>• Costs were not available from all states
**HCUPnet, Healthcare Cost and Utilization Project. Agency for Healthcare Research and Quality, Rockville, MD.**	HCUPnet, Emergency Department, National (U.S.)	The mean charge per emergency stay by age group.:<br><1: Mean Charge per stay: $11,871<br>1-17: Mean Charge per stay: $11,802	N/A	The estimates are from the HCUP National (Nationwide) Emergency Department Sample (NEDS). Total number of weighted visits in the U.S. based on HCUP NEDS was 128,970,364.	<1, 1 - 17	2010	ICD-9-CM Diagnoses Codes:<br>493.00, 493.01,493.02, 493.10,493.11, 493.12, 493.20, 493.21, 493.22, 493.90, 493.91, 493.92, 493, 493.0,493.1, 493.2, 493.9, 493.8, 493.81, 493.82	• The data sets from the HCUP provides both costs and charges in their analysis.<br>• Charges are represented in the direct cost column.<br>• Costs reflect the actual costs of production, whereas charges represent what the hospital billed for the stay.

								• The costs and charges represented are by each discharge and not by each person. If or when calculating costs, the rate of inpatient stays per U.S. children needs to be established to allow the calculation of costs per child.<br>• The HCUP KID, the children only database, was not available for 2010. The costs presented are from the HCUP NIS database. This database divides the under 18 age groups in two, <1, 1-17.<br>• Costs were not available from all states.<br>• There is no national emergency data prior to 2006.<br>• These charges are emergency department (ED) visits in combination with admission to the same hospital, not charges of just a visit to the ED.<br>• This database does not include charges if they visited the ED and were not admitted to the hospital after.
**Agency for Healthcare Research and Quality. Mean expenditure per person with care by condition and event type, United States, 2000. Medical Expenditure Panel Survey.**	MEPS Summary Tables, National (U.S.)	By event, the mean expenditure per person per event type for all ages is:<br>Emergency room visits: $426 (42.0)<br>Home health events: N/A<br>Inpatient stays: $9893 (1,542.5)<br>Office based events: $229 (13.2)<br>Outpatient events: $936 (234.8)<br>Prescription medicines: $229 (8.7)<br>———<br>Categorizes by age as under 18, The total mean expenditure per person is $540. The standard error is greater than 30% and is **199.1.**	N/A	N/A	not specified (all ages), or “under 18.”	2000	Used ICD -9-CM codes related to asthma and categorized internally into “COPD, Asthma”. Under COPD, Asthma, the following is included: chronic obstructive pulmonary disease and bronchiectasis, Asthma Aspiration pneumonitis; food/vomitus, Pleurisy; pneumothorax; pulmonary collapse, Respiratory failure; insufficiency; arrest (adult), Lung disease due to external agents, Other lower respiratory disease, Other upper respiratory disease.	• The MEPS Summary Tables combines Asthma and COPD data in their cost estimates.<br>• This source groups data by event type (emergency room visit, inpatient, etc.) or by age group.<br>• Age group data shows the total mean spending per person and is not separated by event.<br>• Event types include emergency room visits, home health events, inpatient stays, office-based events, outpatient events, and prescription medicines.<br>• Expenditures include payments for medical events reported during the calendar year.<br>• Expenditures in MEPS are defined as the sum of direct payments for care provided during the year, including out-of-pocket payments and payments by private insurance, Medicaid, Medicare, and other sources. Expenditures refer to payments for medical events (office and hospital-based care, home health care, prescribed medicines, dental services, and other medical equipment and services reported during the calendar year).<br>• Payments for over-the-counter drugs and phone contacts with medical providers are not included in MEPS total expenditure estimates.
**Agency for Healthcare Research and Quality. Mean expenditure per**	MEPS Summary Tables, National (U.S.)	The mean expenditure per person for **all ages** by event type is:	N/A	N/A	not specified (all	2010	Used ICD -9 Codes related to asthma and categorized internally into COPD,	• All MEPS Summary Table data will have Asthma and COPD numbers combined.

**person with care by condition and event type, United States, 2010. Medical Expenditure Panel Survey.**	Emergency room visits: $866 (101.4)<br>Home health events: N/A<br>Inpatient stays: $13,570 (2,493.8)<br>Office based events: $430 (24.1)<br>Outpatient events: $1543 (286.0)<br>Prescription medicines: $612 (25.6)<br>(standard error is in parenthesis)<br><br>If we categorize by age as under 18, the total mean expenditure per person is $716.		ages), or "under 18"	Asthma. Under COPD, Asthma, the following is included:<br>chronic obstructive pulmonary disease and bronchiectasis, Asthma Aspiration pneumonitis; food/vomitus, Pleurisy; pneumothorax; pulmonary collapse, Respiratory failure; insufficiency; arrest (adult), Lung disease due to external agents, Other lower respiratory disease, Other upper respiratory disease.	• This source either will allow you to group data by event type (emergency room visit, inpatient, etc.) or by age group.<br>• Age group data will just show the total mean spending per person and will not separate it by event.<br>• Event types include emergency room visits, home health events, inpatient stays, office-based events, outpatient events, prescription medicines.<br>• Expenditures include payments for medical events reported during the calendar year.<br>• Expenditures in MEPS are defined as the sum of direct payments for care provided during the year, including out-of-pocket payments and payments by private insurance, Medicaid, Medicare, and other sources. Expenditures refer to payments for medical events (office and hospital-based care, home health care, prescribed medicines, dental services, and other medical equipment and services reported during the calendar year).<br>• Payments for over-the-counter drugs and phone contacts with medical providers are not included in MEPS total expenditure estimates.

Table S2: Literature Review

Study Reference	Study Name	Geography	Direct Costs	Indirect Costs	Sample Size	Age	Year	How is Asthma Defined	How are Costs Defined and other study notes
**Barrett et al. (2014)**	Trends in Pediatric and Adult Hospital Stays for Asthma, 2000–2010	National (U.S.)	The average cost per hospital stay for children in is $3300 in 2000, and $3600 in 2010.	N/A	The estimates in this Statistical Brief are based upon data from the Healthcare Cost and Utilization Project (HCUP) 2000–2010. Nationwide Inpatient Sample (NIS) and a 2010 disparities analysis file created from the State Inpatient Databases (SID) were used.	Both children from 2-17, and adults.	2000-2010.	Asthma is defined by ICD-9-CM diagnoses codes: 493.00, 493.01,493.02, 493.10,493.11, 493.12, 493.20, 493.21, 493.22, 493.90, 493.91, 493.92, 493, 493.0,493.1, 493.2, 493.9, 293.81,393,82	• Total hospital charges were converted to costs using HCUP Cost-to-Charge Ratios based on hospital accounting reports from the Centers for Medicare & Medicaid Services (CMS).<br>• Costs will reflect the actual expenses incurred in the production of hospital services, such as wages, supplies, and utility costs; charges represent the amount a hospital billed for the case.<br>• For each hospital, a hospital-wide cost-to charge ratio is used. Hospital charges reflect the amount the hospital billed for the entire hospital stay and do not include professional (physician) fees.<br>• Costs are reported to the nearest hundred. Costs were deflated to 2010 using the price index for the gross domestic product.
**Barnett and Nurmagambetov. (2011)**	Costs of Asthma in the United States 2002-2007	National (U.S.)	Per-person cost of asthma per year between 2002 and 2007 inflated for 2009 dollars.<br><br>The total pooled sample reports that the total per person direct cost of asthma ranged from $2,912 to $3,676.	The pooled sample from 2002- 2007 shows that missed school days resulted in a $101 billion dollars.	The study used data from the medical expenditure data survey from 2002 and 2007 and pooled the samples together. The total sample was 206,851 persons. From this sample, 8,719 had asthma, 198,132 did not.	For direct costs, all ages. For indirect costs both adults and children aged 3-19.	2002-2007	• A subject was identified as person with asthma if an (ICD-9-CM) code of 493 was associated with an office-based medical provider visit, a hospital outpatient visit, an emergency department visit, a hospital inpatient stay, or a prescription medication.<br>• Self-reported medical events and prescription medications that were said to be related to asthma were recorded as verbatim text and then converted by professional coders to an ICD-9-CM code of 493 within the data.<br>• The definition for asthma used by this study is utilization based, and hence it does not include persons who reported having asthma as a condition but did not have any asthma-related events or prescription medications during the calendar year	• This study finds the medical and productivity losses due to morbidity and morbidity of asthma at a national level from 2002 – 2007.<br>• Total Expenditures is defined by Office-based medical provider, expenditures, hospital outpatient expenditures, emergency department expenditures, hospital inpatient expenditures, and prescribed medicine expenditures.<br>• The MEPS definition of expenditures is the sum of direct payments for care during the year, including out-of-pocket payments and payments by private insurance, Medicaid, Medicare, and other sources.<br>• Expenditures were chosen as the measurements of cost instead of charges because charges are often discounted and include uncollected liability, bad debt, and charitable care.
**Wang et al. (2005)**	Direct and Indirect Costs of Asthma in School-age Children	National (U.S.)	Total asthma related medical costs in 1996 per child was $401.	Study looks at school absence days, lost productivity arising from parents' loss of time from work and lifetime earnings lost due to premature death of children from asthma: Total indirect	The 1996 MEPS survey was used as the data source. Sample was 2,786 children. Out of the sample, 248 has asthma in 1996.	5-17	1996	The study identified the children with asthma through ICD-9 -CM diagnosis code 493. An event cost associated with an ICD-9 code of 493 was considered an asthma-related medical care cost and was derived from the event files.	• Costs were estimated in 2003 dollars.<br>• For Indirect costs: Published estimates were used for value of lost productivity, and data from the National Vital Statistics System were used for asthma mortality among school-age children.<br>• Expenditures in the 1996 MEPS are defined as the sum of direct payments for care provided during the year, including out-of-pocket payments and payments by private insurance, Medicare, Medicaid, and other sources. For this analysis, the medical events were classified and enumerated into the following mutually exclusive categories: purchase of prescribed medicines,

Study Reference	Study Name	Geography	Direct Costs	Indirect Costs	Sample Size	Age	Year	How is Asthma Defined	How are Costs Defined and other study notes
				cost per child was $**390** per child. Broken down total cost per child for school day absence was **$285** and $**105** for premature deaths.					hospitalizations, emergency room (ER) visits, outpatient hospital visits, office-based visits, other medical equipment, and home health care.
**Nurmagambetov et al. (2017)**	State-level medical and absenteeism cost of asthma in the United States	U.S. States	The Per Person medical cost for childhood asthma ranged from $833 to 1,121.	The total absenteeism cost for childhood asthma ranged from $1.4 Million to $116.5 Million	Used 2008-2012 MEPS files as primary source of Data. They also used restricted access 2003 MEPS, which included state identifiers. Supplemental Data Came from: 2004 National Nursing Home Survey (NNHS), 2005 National Health Accounts, (NHEA), 2000 and 2010 U.S. Census Bureau, 2012 Current Population Survey (CPS), Kaiser Family Foundation 2012 Medicare Beneficiaries data, 2011 Medicaid Statistical Information Statistics (MSIS), the 2014 Bureau of Labor Statistics (BLS) data, and the 2015 Congressional Budget Office (CBO) Long-Term Budget Outlook	0-17	2012	• The study defined a person to have medically treated asthma if the (ICD-9-CM) code 493 was associated with at least one of the following medical events each year: prescription medication, hospitalization, emergency room visit, office-based medical provider visit, or hospital-based outpatient visit.<br>• If they had a diagnosis but no medical treated events – they were defined as to not having asthma. – They define asthma as "Medically Treated Asthma"	This study calculated: per person and total medical costs attributable to asthma for four payer groups (all payers combined and separately for Medicaid, Medicare, and private insurance), Per capita and total number of workdays lost and absenteeism costs attributable to asthma for all ages and for children aged 0 to 17, and Total costs (defined as sum of medical and absenteeism costs).
**Nurmagambetov et al. (2018)**	The Economic Burden of Asthma in the US, 2008 – 2013	National (U.S.)	Annual per-person incremental medical cost of asthma in the United States for ages 0-18 was $1,737.	Per child cost for missed school days is $207, annually.	Used MEPS data from 2008 – 2013 and pooled the samples. The pooled sample was 213,994. From that sample, 10,237 were defined to have asthma.	All ages, with separate estimates for children	2008-2013	• Defines treated asthma as having had at least one medical or pharmaceutical encounter or claim associated with asthma.<br>• Treated asthma was defined as ICD-9-CM diagnosis code 493 (asthma) associated with an office-based medical provider office visit, hospital-based outpatient visit, ER visit, hospital inpatient stay, or filled prescription medication for asthma.	• MEPS data cover expenditures for office-based provider visits, hospital-based outpatient visits, inpatient hospitalizations, emergency room (ER) visits, prescription medications, home health care, dental services, and visual aid.
**(Sullivan et al., 2017)**	The national cost of asthma among school-aged children in the United States	National (U.S.)	In 2015 Adjusted U.S. Dollars:<br>Total - $847<br>Medical - $275<br>ED- $132<br>Inpatient- $554<br>Outpatient- $125<br>Prescription - $360	N/A	The final analytic sample included 22,529 children aged 6 to 11 years (3,118, 13.8% of whom had current asthma) and 21,791 adolescents aged 12 to 17 years.<br>(2,772, 12.7% of whom had current asthma) for a total	6-17	2007-2013	• Asthma is defined by a school aged child who had health care use with the ICD-9 diagnosis code 493.	Expenditures included total, medical, ED, inpatient, outpatient, and pharmacy.

Study Reference	Study Name	Geography	Direct Costs	Indirect Costs	Sample Size	Age	Year	How is Asthma Defined	How are Costs Defined and other study notes
					sample of 44,320 school-aged children.				
**Karaca-Mandic et al. (2012)**	Out-of-Pocket Medication Costs and Use of Medications and Health Care Services Among Children with Asthma	National (U.S.)	The mean annual out-of-pocket asthma medication cost per year was $154 (95% CI, $152-$156) among children aged 5 to 18 years; and $151 (95% CI, $148-$153) among those younger than 5 years.	N/A	The total sample was 8,834 children. 2,921 of whom were younger than 5 years and 5,913 of whom were aged 5 to 18 years.	Under 5, 5-18	1997-2008	• The study identified children with asthma for whom therapy was initiated with a long-acting asthma control medication (inhaled corticosteroids [ICSs], long-acting 2-agonists [LABAs], leukotriene receptor antagonists, combined ICS-LABA formulations, methylxanthines, cromolyn sodium, or immunomodulators) between 1997 and 2007.<br>• The study focused on children requiring control therapy since medication adherence is important to minimizing disease exacerbations and utilization may be related to medication cost sharing.<br>• They restricted their analysis to incident cases of children initiating therapy to study children with newly diagnosed and persistent asthma.	• The obtained data on pharmacy and medical claims from 1997 through 2008 for 37 geographically diverse US employers.<br>• This study is a Retrospective study of insurance claims for 8834 US children with asthma who initiated asthma control therapy between 1997 and 2007. Using variation in out-of-pocket costs for a fixed "basket" of asthma medications across 37 employers, they estimated multivariate models of asthma medication use, asthma related hospitalization, and emergency department (ED) visits with respect to out-of-pocket costs and child and family characteristics.

Table S3: Attached as Excel
